# Brain-computer interfaces and human factors: the role of language and cultural differences—Still a missing gap?

**DOI:** 10.3389/fnhum.2024.1305445

**Published:** 2024-04-11

**Authors:** Cornelia Herbert

**Affiliations:** Applied Emotion and Motivation Psychology, Institute of Psychology and Education, Ulm University, Ulm, Germany

**Keywords:** brain-computer interfaces, language, embodiment, cultural differences, health, EEG, human factors, self relevance

## Abstract

Brain-computer interfaces (BCIs) aim at the non-invasive investigation of brain activity for supporting communication and interaction of the users with their environment by means of brain-machine assisted technologies. Despite technological progress and promising research aimed at understanding the influence of human factors on BCI effectiveness, some topics still remain unexplored. The aim of this article is to discuss why it is important to consider the language of the user, its embodied grounding in perception, action and emotions, and its interaction with cultural differences in information processing in future BCI research. Based on evidence from recent studies, it is proposed that detection of language abilities and language training are two main topics of enquiry of future BCI studies to extend communication among vulnerable and healthy BCI users from bench to bedside and real world applications. In addition, cultural differences shape perception, actions, cognition, language and emotions subjectively, behaviorally as well as neuronally. Therefore, BCI applications should consider cultural differences in information processing to develop culture- and language-sensitive BCI applications for different user groups and BCIs, and investigate the linguistic and cultural contexts in which the BCI will be used.

## 1 Introduction

Brain computer interfaces (BCIs) use brain activity for enhancing the communication and interaction of the user with the environment. Traditional BCI applications focused on vulnerable user groups. These included patients with motoric handicaps or patients with neurological impairments, i.e., users, who by means of BCIs regain a means of self-expression through communication of their thoughts, needs or intentions or through BCI controlled voluntary control of lost actions (for an overview, see, e.g., Luauté et al., 2015). The monitoring of a patient’s mental state, consciousness, motivation or feeling states for brain-computer based interaction is a vibrant and successful field of BCI research. It has continuously grown to include an even broader range of users such as elderly people (e.g., Belkacem et al., 2020), or patients with mental or affective disorders (e.g., Drigas et al., 2020). In addition, several BCIs^1^ have found their way into application domains of healthy users including the occupational or educational setting or BCI use for fun, well-being, recreation or entertainment during leisure time (for a discussion see, e.g., Nijholt et al., 2022).

### 1.1 Human factors in BCI research: language and culture as missing factors

Previous research has impressively shown that BCI performance, and hence, BCI efficiency and BCI literacy are modulated by human factors (e.g., Botte-Lecocq et al., 2014). A variety of human factors has been investigated so far including the user’s satisfaction with the BCI system, the user’s previous experience with technology (for a discussion, see Kübler et al., 2020) or the cognitive demands and mental load associated with BCI use (e.g., Käthner et al., 2014; for review, e.g., Tao et al., 2019). Likewise, the user’s traits or states [e.g., the personality or motivation, current mood, emotion or affect or the cognitive skills (e.g., attention, memory or imagery) of the user] and their impacts on BCI performance have been examined among healthy users or patients. This included well-established BCI systems such as the P300-BCI^2^ (for a recent reviews, e.g., Maslova et al., 2023) or SMR-BCI (for an overview, e.g., Grosse-Wentrup and Schölkopf, 2013). For a joint review of psychological human factors and performance in P300- and SMR-BCIs of healthy and vulnerable BCI users, see Herbert (2024) in the Research Topic.^3^

Although a wealth of human factors have been examined in previous studies, only little is known about how the language of the BCI user and how cultural differences in information processing among BCI users modulate BCI efficiency. The following sections will discuss a number of reasons why these human factors should be examined even more systematically than in previous BCI research (e.g., Grübler and Hildt, 2014).

### 1.2 The language of the user and cultural differences in information processing as BCI research factors for the past and future

The user’s language is of importance in any BCI setting. Every BCI user needs to understand and comprehend the written or orally presented BCI instructions to follow commands or task instructions. Moreover, many BCI applications are using linguistic stimuli for spelling or communication purposes such as the well-known EEG-P300-spellers. Thus, following task instructions or commands or spelling og letters for communication require at least some basic linguistic abilities of the BCI user. Moreover, as outlined below, an impressive number of studies including those from the broader field of human-computer interaction (HCI) suggest that besides the language of the user, cultural differences can shape human behavior, cognition and emotions as well as the subjective experience of the user concerning health and disease or the use of technologies. Therefore, linguistic and cultural aspects and their impact on BCI performance and BCI usage require attention in BCI research and should not be ignored.

The aim of this article is to raise awareness of the relevance of language and cultural differences in BCI research. In line with this aim, the following sections will provide a number of hypotheses and arguments that support the importance of language and of cultural differences as human factors in BCI research. Based on examples from previous research, it is argued that a systematic study of the user’s language, its mental and neural representation and embodied grounding, as well as its relationship with cultural differences of the users could pave the way for the development of linguistic and culturally sensitive BCI applications. Moreover it is proposed that language detection and training are two key topics of enquiry of future BCI studies to improve communication among vulnerable and healthy BCI users. Additionally, it is suggested that embodied language paradigms may provide the unobstrusive assessment of motivational and emotional preferences of vulnerable as well as healthy BCI users. Furthermore, it is suggested that BCI applications should be aware of cultural differences in the perception, processing and evaluation of information to develop culture and language sensitive BCI applications for different user groups and BCIs. This could help support current efforts to move BCI applications beyond the laboratory setting and into the everyday lives of vulnerable and healthy users.

## 2 The role of language in BCI: previous studies and future perspectives

### 2.1 Language comprehension in vulnerable BCI target groups: previous studies

Previously, a number of BCI studies have aimed to determine whether specific even-related brain potentials from non-invasive electroencephalography (EEG), such as the N400 potential (Kutas and Federmeier, 2011) verify as features for the classification of implicit language abilities of the user. Based on the modulation of these brain potentials, assumptions and predictions about mental operations such as semantic understanding of the user could be drawn (for an overview see Dijkstra et al., 2020). Prominent examples of use cases are BCI users, who are mentally or cognitively impaired (e.g., after stroke, traumatic head injuries, or due to progressive neurological disorders), who may additionally suffer from disorders of consciousness (DOC), and who are behaviorally unresponsive and for whom BCI communication may be the only means of interacting with the outside world. This might include patients with locked-in syndrome (LIS; Plum and Posner, 1980) in whom consciousness, awareness and mental functions might be preserved or only partly affected. Several of these studies looked at EEG responses elicited by the presentation of words or sentences to elicit N400 modulation (see Table 1). In addition, a number of studies used auditorily or visually presented questions to test command-following that requires higher-order semantic or syntactic language comprehension or speech recognition abilities. In these studies, the questions had to be answered by the participants by counting the number of yes or no answers flashed on the computer-screen to elicit P300 modulation for target classification (e.g., Huang et al., 2021).

**TABLE 1:** Examples of linguistic paradigms examined for validity in BCI use among vulnerable user groups or healthy subjects to elicit linguistic EEG-ERP modulation, specifically N400 modulation (for details, see the section “The role of language in BCI: previous studies and future perspectives”).

Linguistic paradigms	Users	References
**N400 paradigms (EEG)**	**Patients**	
Higher-order processing including linguistic priming paradigms	Patients with disorders of consciousness (DOC) vs. healthy users	Kotchoubey et al., 2005
Higher-order semantic processing	Patients with disorders of consciousness (DOC)	Schoenle and Witzke, 2004; Steppacher et al., 2013
Semantically related and unrelated spoken word pairs	Comatose patients (with intact temporal lobes)	Rämä et al., 2010
Semantic associative task with congruent or incongruent word sequences (auditory stimuli)	Patients with disorders of consciousness (DOC) and healthy users	Balconi et al., 2013
Cross-study assessment of N400 modulation	Narrative review of N400 effects in disorders of consciousness (DOC)	Wutzl et al., 2021
Assessment of implicit and explicit language abilities with different linguistic paradigms	Systematic review of language abilities, command following and language restoration in patients with disorders of consciousness (DOC)	Aubinet et al., 2022
**N400 paradigms (EEG)**	**Healthy subjects**	
Linguistic paradigms based on EEG-ERP modulation elicited by more complex linguistic reasoning processes such as negation processing: true and false negated statements: N400 and P300/LPP modulation	Healthy subjects, investigation of higher-order semantic processing. The study explored, if paradigms investigating ERP modulation by negated statements verify for use in DOC patients.	Herbert and Kübler, 2011
Sentences with high cloze probability	N400 BCI, healthy users: N400-based anticipation of the sentence endings by the BCI classifiers	Chiossi and Bisiacchi, 2017
Prime (real object)-target (word) pairings, object and word either semantically related or unrelated with the object to elicit semantic concruency-incongruency effects for elicitation of the N400	N400 BCI, healthy users: classification of what might be on the user’s mind during object processing	van Vliet et al., 2010
N400 and brain computer interfacing	A systematic review among BCI user groups	Dijkstra et al., 2020
**EEG databases for semantic concepts in BCI**	**Healthy subjects**	
Study involving six paradigms comprising imagination or perception, and three sensory modalities: visual pictorial, visual orthographic and auditory comprehension. EEG-analysis not limited to specific ERPs such as N400.	Open source EEG dataset (*N* = 12 healthy subjects) for the examination of the neural representation of semantic concepts as input for BCIs in imagination or perception in different sensory modalities.	Wilson et al., 2023

Other studies tried to circumvent higher-order language processing and comprehension, in an attempt to establish BCI-based communication among user groups with probably persistent communicative and language impairments. These patient groups might have a high risk of becoming BCI illiterates in case of insufficient BCI accuracy due to lacking language comprehension. Of these studies, some used a semantic classical conditioning paradigm (e.g., Furdea et al., 2012; Ruf et al., 2013).^4^ The paradigm has yielded accuracy rates of about 65–68.8% (Furdea et al., 2012; Ruf et al., 2013) among healthy users. It was found successful among locked in patients (e.g., Birbaumer, 2006; Kübler et al., 2007), among Alzheimer patients (Liberati et al., 2012), and in one CLIS (completely locked in state) patient with ALS (amyotrophic lateral sclerosis) (De Massari et al., 2013). Further studies used imagery tasks to avoid complex semantic processing. The participants, among them patients with DOC, were verbally instructed to think about two different events eliciting distinct brain signals for classification to answer simple linguistic yes/no questions (for an overview see e.g., Galiotta et al., 2022). Paradigms such as semantic conditioning or those based on mental imagery are not supposed to trigger language-related changes in brain activity. Nevertheless, the users of such paradigms need to be capable of understanding the task, for which instructions have to be provided linguistically.

### 2.2 Future perspectives

Hypothesis 1: Systematic BCI research on human factors related to the language of the user.

Apart from a few exceptions (see above and below) little BCI research appears to be available so far that would have systematically examined human factors related to the user’s language or linguistic competencies for their effects on BCI performance among healthy users and patient populations. Such systematic research however is important to understand if language skills positively impact BCI performance in healthy users and if a lack of these skills might contribute to the high BCI illiteracy and BCI inefficiency rates reported among BCI users in previous studies (for BCI illiteracy, e.g., Edlinger et al., 2015). This hypothesis is underscored by very recent reviews about language abilities in cognitively severely impaired BCI users such as patients with disorders of consciousness (DOC) (see Aubinet et al., 2022). The results imply that residual implicit language abilities (i.e., cortical responses to specific words/sentences) are preserved in about 33–78% of patients with DOCs. Command following using brain-computer interfaces is possible in about 20–50% of DOC patients and language abilities seem to improve during the time course of the rehabilitation.

Moreover, there is evidence that language competencies can be improved by BCI-based training in patients with language or communicative impairments. A number of previous studies provided very promising results in this direction. For example, a recent study by Musso et al. (2022) found faster word processing after brain–computer interface-based language training among stroke patients with mild to severe aphasia. After the training, modulation of event-related brain potentials (ERPs) of aphasic patients accommodated to those of healthy controls. Additionally, detailed linguistic assessment of the participants‘ language abilities showed significant improvement after BCI training beyond spontaneous recovery rates and beyond the trained task (for BCI use in patients with aphasia see also, e.g., Kleih et al., 2016 or for an overview and P300-BCIs, e.g., Fazel-Rezai et al., 2012).

Hypothesis 2: BCI-based language detection and training in vulnerable and healthy BCI users, from bench to bedside to education and the real world.

The observations outlined above support the hypothesis to implement language assessment and language training tools into the BCI applications, particularly for vulnerable target groups. Theoretically, the paradigms included should allow for systematic EEG-based testing of implicit and explicit language abilities. This should include aspects of language comprehension or production on the word, phrase and sentence level, phonology, syntax and morphology, imagery or command-following, respectively. Empirically, as summarized in Table 1, several paradigms from previous studies and reviewed previously (see Aubinet et al., 2022 for DOC or Wilson et al., 2023 for EEG-based BCI datasets of semantic concepts) could provide a good starting point for a standardized BCI language assessment battery. As proposed recently, neurolinguistics provides a rich potential of paradigms based on EEG-ERP modulation that could be used for both, BCI-based language assessment and BCI based language training among vulnerable user groups (for a discussion, Leoni et al., 2021) and that may go beyond N400 modulation (Wilson et al., 2023).

From an application perspective, several user groups with cognitive- or language impairments including behaviorally unresponsive patient groups could benefit from language sensitive BCIs. For example, if comprehension could be detected with a passive BCI system in a particular patient, who might be showing electrophysiological signs of semantic processing (N400 modulation) during language assessment, doctors and staff could be informed about the appearance of changes from one state of consciousness to the other (Aubinet et al., 2022). Next, a hybrid BCI could provide a means of communication and language training in the event that signs of speech and language comprehension or signs of covert consciousness are discovered during the EEG based language examination (for a discussion, see, e.g., Spataro et al., 2022). The BCI paradigms should be linguistically multimodal to reduce barriers, false classification and opt-out of patients due to sensory constraints and expressive restrictions in many of the patient groups.

Brain-computer interface based language training has been conceptualized for applications for a broad range of users and contexts from bench to bedside. In the future this could not only include BCI-based training of language disorders for bedside training or for education in at-risk target groups with language disorders (for reviews Papanastasiou et al., 2020), but include real world BCI scenarios such as BCI-based foreign language learning among emerging adults and children in primary, secondary and/or tertiary education (e.g., Raif et al., 2013; Lekova et al., 2018; Folgieri et al., 2020). In addition, language training by BCIs could include non-invasive methods such as functional near infrared spectroscopy (FNIRS), (e.g., Watanabe et al., 2016).

Methodologically, for real-world BCI applications, it has been suggested that natural language processing combined with artificial intelligence offers great potential for shifting BCI communication from simple spelling and passive language comprehension to more sophisticated applications such as the decoding of continuous speech from cortical semantic representations of healthy users and BCI users from the vulnerable user groups (e.g., Tang et al., 2023; for reviews see, Speier et al., 2016 or Zhao et al., 2023).

Hypothesis 3: Use of embodied language in BCI applications: a potentially unobtrusive alternative to BCI-based action control and decoding of the user’s motivational preferences.

On a neural level, language processing is dedicated to specific language related brain networks (for an overview see, e.g., Friederici, 2011). These language networks may process linguistic information in relative autonomy. Nevertheless, a functional interdependence between language, mental states, perception, action and feelings is put forward by theoretical approaches summarized under the umbrella term of embodiment. From an embodied perspective, mental states are expressed in the body and represented in the brain. Therefore, bodily states (e.g., body postures, gestures, facial expressions, etc.) can modulate how we think and feel in the moment, in the past (memory), and the future (prediction). Therefore, language, cognition, and mental states in general are assumed to be grounded in the body (e.g., Clark, 1998, 2001; Glenberg, 2010; Gallagher, 2011).

The embodiment principle is implicitly underlying many BCI applications; most notably SMR-BCI applications based on motor imagery. Experimentally, moving a device through mental imagery is possible, because as claimed by embodiment theories, in the brain and thus on a neural network level, higher-order cognitive operations such as mental imagination are linked with - lower-level perceptual and motor processes.

**FIGURE 1:**
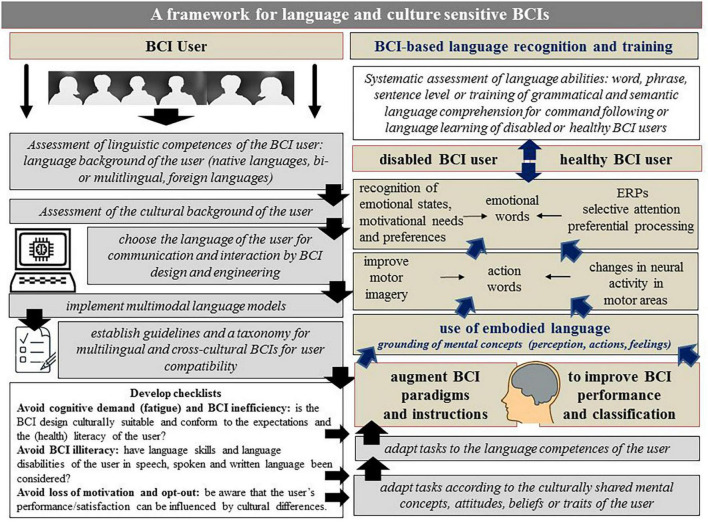
Summary: synopsis of and framework for language and culture sensitive BCIs, see text for details.

Previous BCI studies used the principle of embodiment by augmenting BCI settings with more realistic stimuli such as own body parts imitating bodily actions to facilitate motor imagery of the user in SMR-BCIs (for a discussion, e.g., Serim et al., 2023, for P300-BCIs for an overview e.g., Fazel-Rezai et al., 2012). Some of these studies reported faster or improved BCI performance or stronger feelings of agency, ownership or immersiveness of the users with embodied compared to standard BCI scenarios (e.g., O’Hara et al., 2011; Serim et al., 2023). However, the studies that aimed toward realistic, complex and multimodal embodied BCI scenarios are challenged by technical and user control constraints of BCI systems that use embodied language could be a future alternative future alternative of embodied BCIs. For example, existing BCI systems such as SMR-BCIs could be augmented by the presentation of action words or emotion words. Processing of action words elicits activity changes in distinct areas of the motor cortex, depending on the action that is conveyed by the words (e.g., lick vs. kick). This grounding of action words in the sensorimotor system has been proven by a number of studies many times (e.g., Hauk et al., 2004; for an overview see Pulvermüller, 2013 or Moseley et al., 2015). The processing of action words delivers brain signals that prove discriminant enough for machine learning based feature selection (e.g., Horoufchin et al., 2018). Therefore, as illustrated in Figure 1, action word tasks could improve performance of the user in SMR-BCIs and help translate action word based commands into computer commands for various purposes. Previous EEG studies found that the comprehension of action language modulates oscillatory mu and beta rhythms in the same way as observing actions by watching a video or possibly imagery of that action (Moreno et al., 2013; Klepp et al., 2019).

In addition, a number of emotional paradigms have been proposed for use with different types of BCIs to embody BCIs and classify the user’s mood, preferences or intentions. These studies used paradigms with emotional pictures, scenes, music or videos as stimulus input (for an overview, e.g., Garcia-Molina et al., 2013; Abiri et al., 2019) to induce emotions in the users. Other studies aimed to improve BCI performance, for example during P300-BCI based spelling by additionally using rapid serial visual presentation (RSVP-spellers) with flashing emotional stimuli such as faces (Ron-Angevin et al., 2021). Most recently, datasets comprising hundreds of scenes have been provided to allow pictionary-based communication for the assessment of the physiological needs and motivational states of the user (Proverbio and Pischedda, 2023). RSVP-BCIs equipped with embodied language stimuli such as emotional words could be a promising additional alternative for time-efficient BCI-based communication of motivational and emotional preferences of the users in real world BCI applications. A particular advantage of using words for conveying emotions is their power to significantly modulate - akin to picture stimuli (Schupp et al., 2006) - early and late event-related brain potentials across several runs of stimulus repetitions without significant habituation when presented in RSVP (rapid serial visual presentation), e.g., Kissler et al., 2007; Herbert et al., 2008.

The seemingly endless vocabulary of languages, the existence of normative word corpora for emotional words in many languages (e.g., Buechel et al., 2020), the emotional modulation of EEG activity by visually presented words as well as emotion recognition by emotional prosody and speech during the presentation of auditory stimuli (e.g., Paulmann et al., 2013; for a review Jaswal et al., 2022) might offer enormous potential for assessing the BCI user’s preferential processing of motivational and emotional states across languages.

Hypothesis 4: Mastering language diversity in BCI use.

Moreover, language is characterized by an unlimited diversity. According to current estimates 6,500 languages are spoken in the world (Comrie, 2002; Pereltsvaig, 2020). Similarly, a considerably number of the population world-wide is raised bi- or multilingual or learning a second foreign language early or later during life with considerably proficiency (e.g., Ivana, 2022). The market of BCI applications (e.g., in healthcare, smart home control, communication, entertainment and gaming) is growing. Thus, a growing number of user populations from countries all over the world (see “Brain Computer Interface Market” Research Report 2023–2031) will be using a BCI. BCI technology is still lagging behind these trends. As pointed out previously, most language implementations in BCIs are still restricted to a single language, primarily the English language (Speier et al., 2016). As reported by Loizidou et al. (2022), the traditional P300-BCIs for example have been trained in a few languages (e.g., German, English, Dutch, or Chinese) and among native speakers only. Therefore, recent studies as the one by Loizidou et al. (2022) aimed to include multilingual language models to compare spelling accuracy among healthy users with different native language backgrounds including users fluent and proficient in more than one language (e.g., Greek, Spanish vs. English, and English being the second fluent language).

Regarding bi- or multilingual BCI users, there is evidence of significant differences in the functional organization of the brain in bilingual vs. monolingual individuals (for an overview, Bialystok, 2017). For example there are findings about differences between bilingual vs. monolingual individuals in cognitive functions such as attention, memory, or prefrontal executive functions, and of lower risk or different recovery from stroke. In addition, there are findings suggesting different neuronal patterns of activation among bilingual vs. monolingual individuals in a number of paradigms related to BCI performance such as imagery, semantic conditioning, or word fluency (e.g., Blom et al., 2017; Grégoire and Greening, 2019; van Dijk et al., 2019; Berkes et al., 2020). Moreover, previous studies found that mental imagery varies as a function of the language of the user (native language vs. second, or foreign language, e.g., Vigliocco et al., 2005; Hayakawa and Keysar, 2018; for alternative explanations, see Montero-Melis et al., 2020). Moreover, there is evidence that training of mental imagery can improve performance in language processing (for a discussion, see, e.g., Bayram et al., 2023). This again supports the hypothesis 1 outlined earlier in this section about transfer effects of BCI training and language. BCI-based training effects may have benefits for other cognitive functions that were not specifically trained during the BCI session.

Therefore, as illustrated in Figure 1, having the option to choose the preferred language for BCI-based communication may psychologically encourage many BCI users to reach their desired BCI performance. Speaking, acting, and interacting in the language of choice may help BCI users feel less fearful and more confident in their mastery of the technology. Recent research has shown that the two motivational factors of incompetence fear and mastery confidence as assessed via standardized questionnaires are important motivators that affect how well healthy and vulnerable BCI users perform with various types of BCIs (e.g., Nijboer et al., 2008, 2010; for an overview e.g., Herbert, 2024). Technological advancements in the field of within- and cross-language brain decoding are favorable to the endeavor of providing multi-language sensitive BCIs. The field of language decoding has moved from a focus on decoding words and concrete concepts from brain activity patterns in one language to the use of more naturalistic experimental tasks that involve higher-level language processing including discourse processing. Moreover, computational modeling allows the translation of one language into another second language (Xu et al., 2021). While imaging techniques have been the focus of most previous decoding studies, recent research shows that machine learning and non-invasive EEG methodology can achieve promising results in cross-language decoding as well (Foster et al., 2021). In addition, the user’s languae skills, such as fluency in multiple languages, should be considered in the context of observations about cultural differences in information processing (see Hypothesis 5). Being raised in and speaking more than one language may mean being immersed in more than one culture. Moreover, language use may shape the minds of the speakers (e.g., Ramírez-Esparza and García-Sierra, 2014; Kroll et al., 2018). The cultural background of the user determines the user’s native language(s) and the language(s) in which the user can communicate with the BCI system.

Hypothesis 5: Considering cultural differences and BCI use.

Given the mission of BCI research to treat every user regardless of age, gender or language, it must be recognized that even very basic and fundamental mental processes and their respective neural correlates are shaped by the culture and socio-cultural context of an individual (for overviews, e.g., Ambady, 2012; Kitayama et al., 2019). Theoretically, culture is a multifaceted concept. Its investigation may encompass the study of culturally constructed norms and beliefs and how socio-cultural contexts modulate individual experience, behavior, affect and cognition, or a person’s cultural identity, personality and self-concept (e.g., see APA Dictionary of Psychology^5^). Cultural differences manifest particularly at the level of information processing and in how information is perceived, processed, and evealuated. Cultural differences can be measured at the level of the brain based on different modulations of, for example, neuronal activity in response to certain stimuli, as well as at the level of subjective experience and behavior (e.g., for review Markus and Kitayama, 1991; Park and Huang, 2010; Han and Ma, 2014).

Thus, as outlined in detail below and as summarized in the synopsis and framework in Figure 1, one can think of at least three important ways of how cultural differences in information processing could modulate BCI performance and BCI use. First, by modulating perception and action. Second, by modulating verbal and non-verbal communication e.g., of emotions and feelings. Third, by shaping personality and beliefs about technology, health and disease.

Observations from behavioral neuroscience about cultural differences in information processing are a prominent example illustrating the impact of cultural factors on perception, action and emotions. The studies suggest that cultural differences in information processing exist in the representation of basic concepts such as those of time, body and space including basic spatial reference frames such as the meaning of up vs. down or left vs. right, relevant for mental imagery paradigms used in previous SMR-BCIs studies. Moreover, the perspective from which mental representations on body movements and actions are imagined (i.e., either from an egocentric first person perspective or a third person perspective) generally can differ between cultures (e.g., Bohnemeyer et al., 2014). Cultural differences and how they can impact information processing and user interface design are well recognized topics in the field of human-computer interactions (HCI). Thus, HCI design guidelines take into account peculiarities in the use of different languages (e.g., Rau et al., 2012) and in spatial information and reference structure. This is done to meet the different and culturally modulated expectations of the user and to allow optimal ergonomic fit of graphical user interfaces and information architectures for different user groups with different language and cultural backgrounds (for an overview, e.g., Plocher et al., 2021). Therefore, guidelines for cross-cultural design from the broader domain of HCI could be a good starting point for cross-cultural BCI engineering as well (e.g., Plocher et al., 2021). These cross-cultural design guidelines from HCI research incorporate insights from cross-cultural psychology to ask how to design and construct user interfaces that are responsive to cultural differences in perception (e.g., layout, format) or cognitive-linguistic aspects of users with different cultural backgrounds. The guidelines also aim to take into account cultural differences of the user in attitudes, values and preferred communication style, that can affect user performance and the usability of technology.

For example, regarding communication and expression of emotions and the ability to understand and share the feelings of another, such as empathy, these human factors are modulated by cultural differences and have been shown to modulate BCI performance in previous BCI studies using P300- or SMR-BCIs (Kleih and Kübler, 2013; Hammer et al., 2014; Kleih-Dahms et al., 2021). The differences go beyond just contrasting the main individualistic and collectivistic cultures. As evidenced by a recent study on the processing of gestures in French versus Italian, cultural differences can also relate to variation in brain responses and inter-brain connectivity among individuals thought to have subtler cultural differences in the understanding of emotional states (e.g., Balconi and Fronda, 2022). These differences may furthermore extend to speakers with varying sociolinguistic backgrounds and linguistic proficiency.

Moreover, previous BCI studies have observed that sociodemographic factors, including the gender of the user or experimenter, as well as broader social factors may influence BCI performance (Zich et al., 2017; Pillette et al., 2021; for review of social factors, e.g., Sexton, 2015). In line with this, cross-cultural studies suggest that cultural differences can modulate personality, beliefs about technology, health and disease. Regarding personality, cultural differences have been found for a number of personality traits whose impact on BCI performance has been investigated in previous BCI studies among healthy and vulnerable BCI user groups, most notably the Big Five Personality traits (e.g., Leeuwis et al., 2021; for overviews, e.g., Herbert, 2024). These differences in personality measures may further vary in bilingual individuals (see Hypothesis 4) who change their personality as they switch between their two languages and/or cultures (Dylman and Zakrisson, 2023).

Cultural differences and language-related differences may additionally apply to the perception of self-success and failure, and to beliefs about and acceptance of technology and mental ill health (Sheikh and Furnham, 2000; De Vaus et al., 2018; Salvador et al., 2022). Overall, in the context of BCI use, these cultural differences could affect the BCI user’s intrinsic motivation (mastery confidence), empathy, trust in and affinity toward BCI technology, the user’s well-being, sense of agency and ownership as well as the user’s appraisal of what and which stimuli are self-relevant. All of these human factors have been suggested to significantly affect BCI performance for example in the P300 speller or the SMR-BCI (e.g., Nijboer et al., 2010; Kleih and Kübler, 2013; Kleih-Dahms et al., 2021; for an overview Herbert, 2024). Furthermore, cultural differences influence feelings of sense of self, agency and ownership by determining how the self is appraised in relation to others, society, and nature (for meta-analysis e.g., 234 Han and Ma, 2014). This cultural shaping could influence the perceptual and somatosensory motor experiences of the BCI user. Therefore, cultural differences are of relevance for understanding differences among users of a BCI in the grounding of mental concepts in bodily experiences (perception, action, sensation, and feelings) (e.g., Kövecses, 2010) that may modulate BCI performance in P300-BCIs or SMR-BCIs (see Hypothesis 3). Regarding self-relevance, a paradigm frequently used in BCI research for assessing self-referential processing in patients with DOC is the subject’s own name paradigm (SON) (Perrin et al., 2006; Laureys et al., 2007; Kempny et al., 2018). Neuroscientific studies suggest considerable cultural variability in the brain correlates elicited in the SON and of whether self-referential stimuli are processed preferentially in comparison to other-referential stimuli (e.g., Zhu et al., 2007; Shi et al., 2011; for overviews see also Han and Northoff, 2009; Han and Humphreys, 2016). The examples discussed above are just one of several examples that illustrate how cultural differences can implicitly influence BCI outcome measures. If they are ignored, this could lead to wrong assumptions about the BCI performance of the users and impair the prediction of the severity of symptoms of vulnerable BCI users. such as patients with DOC.

## 3 Conclusion

This hypothesis and theory paper aimed at raising awareness for including the user’s language and cultural differences as human factors in BCI research. As explained in the previous sections, an interdisciplinary, theory-driven exploration of linguistic and cultural factors and their relevance to design and engineering aspects has not yet been established as a fundamental part of a user-centered BCI approach (for discussion, see, e.g., Kübler et al., 2015 and recent trends e.g., Gena et al., 2023). Therefore, future BCI applications should particularly draw attention to linguistic and cultural aspects when designed to include various users. Knowing that BCI instructions as well as many paradigms used in BCI applications are language- and culture-dependent may pave new ways for language and culture sensitive BCI engineering with respect to input (paradigms and tasks for training, feature extraction) and output (classification and application interface) in harmony with the individual user’s linguistic and cultural background. In the human brain, basic processes of perception, action, emotions and communication are not hard-wired but adapt their functionality to the cultural and sociolinguistic context. This supports the need to include language and cultural factors more systematically in the research, paradigms and the design of BCIs. As summarized in Figure 1, in future BCI studies, linguistic and cultural variations in the perception, processing, and evaluation of stimuli in BCI paradigms could be achieved, for example, by expanding the stimulus database. Representational differences between languages and cultures in concepts such as time, body, action, or reference space may require the design of BCI interfaces for culturally heterogeneous users, consistent with existing cross-cultural design guidelines and recommendations from the broader field of human-computer interaction research.

## Data availability statement

The original contributions presented in the study are included in the article/supplementary material, further inquiries can be directed to the corresponding author.

## Author contributions

CH: Writing – original draft, Writing – review & editing, Conceptualization, Investigation, Methodology, Validation, Visualization, Formal analysis, Project administration, Resources.

## Footnotes

1 BCI systems can be classified as active, reactive or passive. Active and reactive BCIs allow immediate and synchronous interaction of the user with the environment, whereas passive BCIs provide a tool for the monitoring of the user’s mental state by means of brain activity without active engagement of the user (for an overview, e.g., Zander et al., 2010). Moreover, hybrid BCIs (e.g., Pfurtscheller et al., 2010) may use more than one brain signal and comprise an interface that allows switching from one target signal to the other.

2 Some BCIs have been used intensively, especially among vulnerable target groups for use in the clinical setting (e.g., neurorehabilitation). Among those, are BCIs that are using motor imagery (MI) as a paradigm or task to elicit changes in the sensorimotor rhythm (SMR) of the brain for immediate BCI based control of certain devices by the user (in short: SMR-BCIs or MI-BCIs). Similarly frequent are BCIs that are using flickering visual stimuli for eliciting steady state visual or auditory stimulations (SSVEP-BCIs). Finally and most traditionally used, are BCI systems for spelling such as the well-known P300 spellers (for an overview e.g., Edlinger et al., 2015). These elicit discrete event-related brain potentials (ERPs) based on the voluntary attention of the user and the user’s choice of numbers, letters or symbols rapidly presented in rows and columns.

3
https://www.frontiersin.org/research-topics/31277/analyzing-and-computing-humans—the-role-of-language-culture-brain-and-health

4 During semantic classical conditioning, statements that are immediately rewarded or punished by an unconditioned stimulus (US) are presented to establish a conditioned response (CR) between the verbal input (sentences/statement) and the US. The advantage of semantic classical conditioning for BCI based communication is that not the original semantic or mental state is to be distinguished by the BCI classifier but the learned emotional state associated with the verbal statement (Birbaumer et al., 2012; Liberati et al., 2012).

5
https://dictionary.apa.org/culture
